# A multicenter prospective, randomized, placebo-controlled phase II/III trial for preemptive acute graft-versus-host disease therapy

**DOI:** 10.1038/s41375-020-01059-3

**Published:** 2020-10-20

**Authors:** Eva M. Weissinger, Jochen Metzger, Michael Schleuning, Christoph Schmid, Diethelm Messinger, Gernot Beutel, Eva-Maria Wagner-Drouet, Johannes Schetelig, Herrad Baurmann, Andreas Rank, Friedrich Stolzl, Kerstin Schäfer-Eckart, Karin Westphal, Wolfgang Bethge, S. von Harsdorf, Donald W. Bunjes, Daniela Heidenreich, Stefan Klein, Ernst Holler, Hans H. Kreipe, Danny Jonigk, Irina Türüchanow, Julia Raad, Armin Papkalla, Heiko von der Leyen, Lothar Hambach, Iyas Hamwi, Steve Ehrlich, Jurgen Krauter, Michael Stadler, Arnold Ganser

**Affiliations:** 1 Department of Hematology, Hemostasis, Oncology and Stem Cell Transplantation, Hannover Medical School, Hannover, Germany; 2 Mosaiques Diagnostics GmbH, Hannover, Germany; 3 Deutsche Klinik fuer Diagnostik, Wiesbaden, Germany; 4 Klinikum Augsburg, Augsburg, Germany; 5 Prometris, Mannheim, Germany; 6 III. Department of Medicine—Hematology, Internal Oncology & Pneumology, Johannes Gutenberg-University Medical Center, Mainz, Germany; 7 Technical University Dresden, Dresden, Germany; 8 DKMS GmbH, Dresden, Germany; 9 Helios Klinikum Berlin-Buch, Berlin, Germany; 10 Klinikum Nürnberg, Paracelsus Medizinische Privatuniversität, Nuremberg, Germany; 11 University of Tuebingen Medical Center, Tuebingen, Germany; 12 Department of Internal Medicine III, University of Ulm, Ulm, Germany; 13 Department of Hematology and Oncology, University Hospital Mannheim, Heidelberg University, Heidelberg, Germany; 14 Department of Internal Medicine III, University Hospital Regensburg, Regensburg, Germany; 15 Institute of Pathology, Hannover Medical School, Hannover, Germany; 16 Hannover Clinical Trial Center (HCTC), Hannover Medical School, Hanover, Germany; 17 Department of Internal Medicine III, Municipal Hospital of Braunschweig, Braunschweig, Germany

**Keywords:** Translational research, Clinical trials

## Abstract

Acute graft-versus-host disease (aGvHD) contributes to about 50% of transplant-related mortality (non-relapse mortality) after allogeneic hematopoietic stem cell transplantation (HSCT). Here the predictive value of a urinary proteomic profile (aGvHD_MS17) was tested together with preemptive prednisolone therapy. Two-hundred and fifty-nine of 267 patients were eligible for analysis. Ninety-two patients were randomized upon aGvHD_MS17 classification factor above 0.1 to receive either prednisolone (2–2.5 mg/kg, *N* = 44) or placebo (*N* = 47; *N* = 1 randomization failure) for 5 days followed by tapering. The remaining 167 patients formed the observation group. The primary endpoint of the randomized trial was incidence of aGvHD grade II between randomization and day +100 post HSCT. Analysis of the short-term preemptive prednisolone therapy in the randomized patients showed no significant difference in incidence or severity of acute GvHD (HR: 1.69, 95% CI: 0.66–4.32, *P* = 0.27). Prednisolone as preemptive treatment did not lead to an increase in relapse (20.2% in the placebo and 14.0% in the prednisolone group (*P* = 0.46)). The frequency of adverse events was slightly higher in the placebo group (64.4% versus 50%, respectively). Taken together, the results of the Pre-GvHD trial demonstrated the feasibility and safety of preemptive prednisolone treatment in the randomized patients.

## Introduction

Acute graft-versus-host disease (aGvHD) is a severe complication of allogeneic hematopoietic stem cell transplantation (HSCT) and is diagnosed by clinical features, such as skin rash, diarrhea, or elevation of liver enzymes followed by biopsies and histopathological examination if appropriate [1]. Between 30 and 80% of patients develop aGvHD, depending on primary disease, patient age, conditioning regimen, and GvHD prophylaxis [2–4]. Initial standard therapy for aGvHD is prednisolone at a dose of 2–2.5 mg/kg body weight per day, resulting in a response rate of about 70% for patients with aGvHD grades I–II [4–6].

Proteome analysis of urine using capillary electrophoresis coupled online to mass spectrometry (CE–MS) to define differentially excreted urinary peptides is a broadly applicable and powerful diagnostic tool in a variety of diseases [7–10]. The proteomic classifier “aGvHD_MS17” was based on 17 differentially excreted peptides identified by CE–MS analysis in the urine of patients after allogeneic HSCT. Upon application of support vector machine (SVM)-based analyses tools, the dimensionless classification factor (CF) for aGvHD_MS17 was calculated [11]. Receiver-operated characteristic curves were used to determine that a CF above 0.1 could be used to separate patients with pending aGvHD grades II–IV from those who never developed aGvHD or had aGvHD grade I up to 21 days prior to clinical manifestation of aGvHD [11–13]. The aGvHD_MS17 classifier has been tested on more than 700 patients transplanted at four different transplant centers. Apart from the aGvHD_MS17 profile, others have described the use of ELISA to detect plasma biomarkers, to predict aGvHD outcome and non-relapse mortality (NRM) [14–16]. Bacigalupo et al. [17] published the first preemptive treatment trial for aGvHD, using ATG in all patients stratified into three risk groups and resulting in a reduction of acute and chronic GvHD and better overall survival (OS) in the high-risk subgroup. The same group studied the influence of steroid treatment in patients with aGvHD grade I randomized to receive 1 mg prednisolone or placebo to prevent severe GvHD [18]. Based on our previous studies, we initiated a German multicenter trial in 2008, to evaluate the effect of preemptive prednisolone treatment of imminent aGvHD grades II–IV upon aGvHD_MS17 CF positivity. The trial was an investigator initiated, prospective, multicenter, placebo controlled, double-blind clinical trial, registered under www.isrctn.com: ISRCTN03911524 and www.clinicaltrialsregister.eu: EudraCT number: 2008–005862–30.

## Methods

### The PRE-GvHD trial

The “Pre-emptive therapy of aGvHD according to specific proteomic patterns after allogeneic hematopoietic stem cell transplantation (Pre-GvHD) trial” was an investigator initiated, prospective multicenter, double-blind, placebo controlled, randomized phase II/III trial conducted in 11 German centers. The trial was designed and overseen by the authors. The trial protocol was approved by the leading ethics committee of Hannover Medical School (MHH), those of the contributing sites and the federal regulatory agency (BfArM). The trial was performed in accordance with the principles of the Declaration of Helsinki.

### Patients

**Fig. 1:**
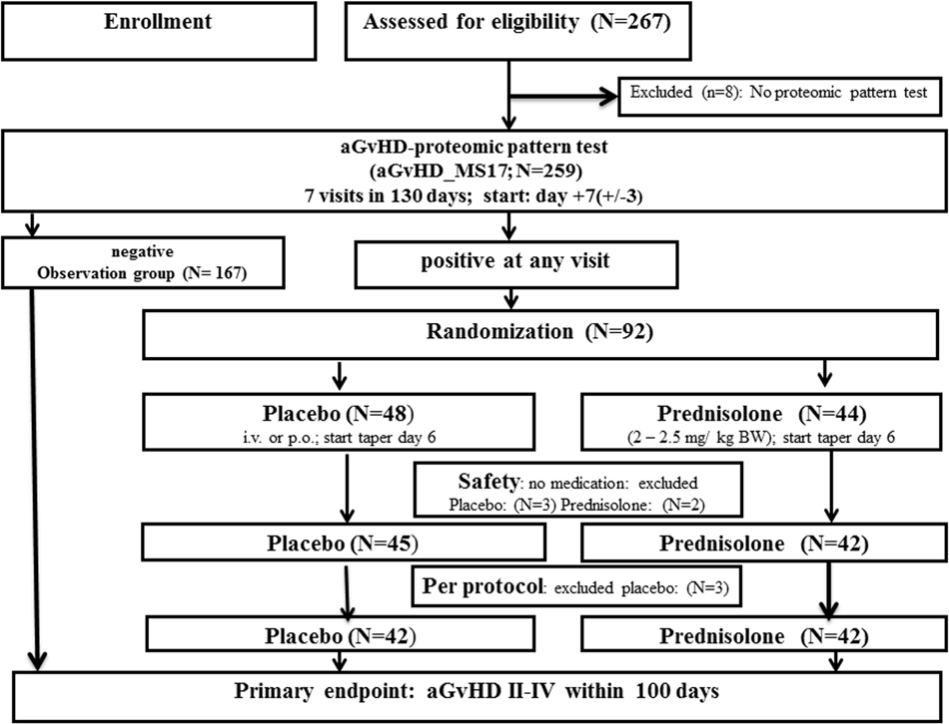
Trial flow chart. Patients (*N* **=** 267) were enrolled in the PRE-GVHD trial after informed consent and 259 were eligible. The screening phase was from day 0 to day +3 and the first samples were collected and analyzed on day +7 (±3). Upon aGvHD_MS17 CF (CF > 0.1) positivity, patients were randomized to receive either prednisolone or placebo for 5 days followed by 19 days of tapering. Primary endpoint was aGvHD grades II–IV. Without clinical manifestation of aGvHD, the medication was be tapered on days 6–19 after initiation of the therapy. Patients with aGvHD_MS17 negative samples were continually monitored until either a sample was positive for the aGvHD_MS17 CF, when they were randomized, or until clinical manifestation of aGvHD. Upon clinical manifestation of aGvHD, the patients were treated with standard therapy for aGvHD and counted as “pattern failure.” Eight patients were excluded due to missing urine samples and proteomic tests. The observation group consisted of 167 patients. Patients (*N* = 92; ITT population) were randomized to either the placebo (*N* = 48) or prednisolone (*N* = 44) arms. Fife patients (*N* = 3 placebo and *N* = 2 prednisolone) did not receive the study medication and were excluded from the safety group (*N* = 87) and safety analyses. The per-protocol population (*N* = 84) excluded another three patients from the placebo group who either received the study medication for <3 days (*N* = 2) or had no positive proteomic pattern test (*N* = 1).

Adult patients (from 18 years on) were included after informed consent. Inclusion was on day +3 (range: 2–5) after the first allogeneic HSCT. Detailed inclusion and exclusion criteria are provided in Supplementary material. The first patient entered the trial on December 14, 2009. Eight patients were excluded from analysis, since no urine samples were collected and analyzed, due to intensive care treatment, dialysis or death prior day +7. Clinical and demographic data for all eligible patients are summarized in Table 1. Urine was collected at regular predefined intervals up to 80 days post HSCT and analyzed centrally. Ninety-two patients were randomized upon aGvHD_MS17 CF positivity to receive either prednisolone (2–2.5 mg/kg, *N* = 44) or placebo (*N* = 48) for 5 days followed by a taper of 19 days in the absence of aGvHD. One patient of the placebo group was randomized by mistake despite only negative samples in the aGvHD_MS17 test. The remaining 167 patients formed the observation group. Clinically manifest aGvHD was graded according to the modified Glucksberg criteria [19] and verified by biopsies [20] where appropriate. Visits were made weekly until day +35 and on days +50, +80 (all ±3 days), +100, and +130 (±10 days) for clinical evaluation and development of aGvHD. The follow-up was 1 year after HSCT. Figure 1 shows the trial flow chart and the disposition of patients (Supplementary Table 1).

**Table 1:** Patient characteristics.

Patient characteristics	Placebo *N* = 48 (%)	Prednisolone *N* = 44 (%)	Observation *N* = 167 (%)
Age	58 (19–73)	55 (21–70)	52 (19–74)
Gender			
Male	27 (56)	29 (66)	109 (66)
Female	21 (44)	15 (34)	58 (34)
Primary disease			
Acute (AML, ALL, sAML)	25 (52)	24 (55)	92 (55)
Chronic (MDS, MPS, CML, CLL)	12 (25)	10 (23)	42 (25)
Lymphoma (NHL, HD,MM)	10 (21)	9 (20)	31 (19)
Nonmalignant (AA, PNH)	1 (2)	1 (2)	2 (1)
Status primary disease			
CR 1/CP1	17 (35)	21 (48)	82 (49)
CR 2 or higher	5 (10)	5 (11)	20 (12)
No CR (untreated, relapse, refractory)	25 (52)	17 (39)	58 (35)
Vo status	1(2)	1 (2)	7 (4)
Conditioning			
Myeloablative (MAC)	9 (19)	13 (29.5)	40 (24)
Reduced intensity conditioning (RIC)	39 (81)	31 (70.5)	127 (76)
Graft			
PBSC	43 (90)	38 (86)	155 (93)
BM	5 (10)	6 (14)	11 (6.5)
Other	–	–	1 (0.5)
Immunosuppressive antibodies			
None	12 (25)	10 (23)	51 (31)
ATG Fresenius	29 (60)	30 (68)	107 (64)
Thymoglobulin	2 (4)	1 (2)	4 (2)
Campath	3 (6)	3 (7)	6 (4)
GvHD prophylaxis			
CSA/MTX	18 (37)	14 (32)	73 (44)
CSA/MMF	21(44)	25 (57)	73 (44)
Other	9 (19)	5 (11)	21 (12)
Donor			
Related	9 (19)	13 (30)	39 (23)
Unrelated	39 (81)	31 (70)	128 (77)
HLA match			
Matched	43 (90)	36 (82)	146 (87)
Mismatched	5 (10)	8 (18)	21 (13)
Gender (R/D)			
Mismatched (male/female)	6 (12.5)	9 (20.4)	25 (15)

RIC protocols (*N* = 203) consisted of fludarabine (Flu), amsacrine, AraC, and TBI or busilvex (FLAMSA [25]; *N* = 31/203; 15%); BNCU–Flu–melphalane (BFM; *N* = 27/203; 14%), Flu–Bu (*N* = 34/203; 17%); Flu–Mel (*N* = 34/203; 17%); Flu–treosulfane (Flu–Treo; *N* = 16/203; 8%); total body irradiation (TBI)–Flu (*N* = 16; 8%); and other (*N* = 42; 21%).

Acute: *AML* acute myeloid leukemia, *ALL* acute lymphatic leukemia, *sAML* secondary AML; chronic: *MDS/MPS* myelodysplastic/proliferative syndrome, *CML* chronic myeloid leukemia, *CLL* chronic lymphatic leukemia; lymphoma: *NHL* non-Hodgkin lymphoma, *HD* Hodgkin disease, *MM* multiple myeloma; nonmalignant: *SAA* severe or very severe aplastic anemia, *CR/CP* complete remission/chronic phase; *no CR* untreated, relapse, refractory, *MAC* myeloablative conditioning, *RIC* reduced intensity conditioning, *PBSC* peripheral blood stem cells, *BM* bone marrow, *CB* cord blood, *ATG* anti-thymocyte globulin, *CSA* cyclosporine A, *MTX* methotrexate, *MMF* mycophenolate motefil; other: MMF, tacrolimus (FK506), or different combinations of immunosuppressants.

### Urine sample collection

Samples were collected from all patients enrolled weekly between days +7 and +35, on days +50 and +80 (all: ±3 days) after HSCT, frozen at −20 °C and shipped to the central laboratory (Mosaiques, Hannover, Germany) for further analysis. The protocol required that the time between sample arrival at Mosaiques and the transmission of the aGvHD_MS17 CF score for randomization had to be within 72 h. Sample preparation and CE–MS analyses were described previously and summarized in Supplementary material [21].

### Blinding and randomization procedures

All patients included in the screening process received a consecutive patient number at their study site. The patient identifier was the combination of the study site number and the patient number. For patients not eligible for the trial, only demographic data and inclusion/exclusion criteria were recorded. Two independent teams at each study site ensured double-blinding. Team A (clinical ward team) received only blinded medication and did not know the treatment code. Team B (pharmacy) knew the treatment code prepared by the independent trial statistician and prepared the medication accordingly. Randomization lists were prepared by the statistician at Prometris (Mannheim, Germany) who was not involved in other tasks of the trial. Randomization envelopes containing the treatment code were deposited at each study site, accessible only for team B. Additionally, sealed emergency envelopes containing the randomization number and the treatment group was prepared for each patient to be opened in case of signs of aGvHD grades II or higher or another medical emergency.

### Primary and secondary endpoints of the clinical trial

The primary endpoint was the occurrence of aGvHD grades II–IV between randomization and 100 days after HSCT. Death occurring in patients without aGvHD (grade II or higher) between randomization and 100 days after HSCT was considered as treatment failure, equivalent to aGvHD grade II. The secondary efficacy endpoints were (1) the severity of aGvHD between time of randomization and 100 days after allo-HSCT, (2) occurrence of aGvHD grades II–IV, (3) severity of all aGvHD, (4) transplant-related mortality (NRM), (5) OS, (6) occurrence of leukemic relapses, and (7) infectious complications.

### Safety

The exposure of study treatment was characterized by the number of administrations and the cumulative dose for both treatment groups. Adverse events (AEs) were coded using the Medical Dictionary for Regulatory Activities, version 19.1. Annual Safety Reports were submitted to National Competent Authority and leading ethic committee as required by ICH guidelines and national regulations.

### Statistical analysis

Primary endpoint of the Pre-GvHD trial was incidence of aGvHD grades II–IV within 100 days post HSCT. Sample size calculation was based on previous data [22]. We expected that aGvHD_MS17-positive samples would predict 80% probability of developing aGvHD grades II–IV and a reduction to 41% by preemptive therapy (pilot study, Supplementary Table 1a; [22]). Seventy-eight patients (2 × 39) were required to detect a reduction of incidence or severity of aGvHD grades II–IV or death from 80% in the placebo group to 50% (odds ratio: 0.3) in the prednisolone group with a type I error of 5% and a power of 80% using a two-sided Cochran–Mantel–Haenszel (CMH) test. The number of patients for randomization was increased to 2 × 45 to account for dropout. A detailed description of the statistical plan is provided in the Supplementary materials. The CMH test and competing risk analysis were used to compare the two treatment arms for incidence of aGvHD II–IV within 100 days post HSCT. OS in both treatment arms was compared using Cox proportional hazard models with left truncation at time of randomization and right censoring in case of lost to follow-up. Time dependent Cox models were used to compare OS between all randomized patients with samples positive for the aGvHD_MS17 test versus all patients in the observation group with continuously negative aGvHD_MS17 test results.

## Results

### Patient characteristics

Between 2009 and 2015, 267 patients were enrolled into the pre-randomization phase of the Pre-GvHD trial after the first HSCT; 259 patients were eligible for assessment of outcome. The disposition of patients and trial flow diagram are shown in Fig. 1 (Supplementary Table 1). Urine samples were monitored at indicated time points and the aGvHD_MS17 CF was calculated by application of an SVM-based software [21, 23] and considered positive when the CF was above 0.1 [11, 24]. The mean time of arrival of the sample at the central laboratory and transmission of the aGvHD_MS17 CF results for randomization was 30.1 h (95% CI: 29.4–30.8). All patients were continually monitored until sample positivity when they were randomized or, in case of negative samples, until day +80 (±3). Patients were not randomized, if aGvHD_MS17 CF was negative at the visit, or if exclusion criteria for preemptive treatment were met at time aGvHD_MS17 CF positivity (Supplementary material). The follow-up was 1 year after HSCT. Patient characteristics are summarized in Table 1. The majority of the patients (70%) were transplanted in complete remission (CR), received immunosuppressive antibodies (placebo: 75%, prednisolone: 84%, and observation: 78%), were transplanted from matched unrelated donors using reduced intensity conditioning regimens (RIC, placebo: 81%, prednisolone: 71%, and observation: 76%), and a calcineurin-inhibitor-based GvHD prophylaxis (CsA) in combination with either methotrexate or mycophenolate mofetil (Table 1). There was a difference in HLA (10 versus 18%) and gender mismatch (12 versus 20%) in the placebo compared to the prednisolone arm. The observation group consisted of 167 patients, 65 of those had samples scoring positive for aGvHD_MS17 CF but were not randomized for various reasons summarized in Table 2. Forty-one patients developed aGvHD I–IV (63%) and four patients (6%) died prior to day 100. Eighteen patients had positive samples prior to manifestation (clinical onset) of aGvHD I–IV or death (median: 30 days; mean: 14 days; range: (7–77), while in 25 patients clinical manifestation was earlier (median: 6 days, mean: 1 day; range: −3 to 42). Reasons for no randomization are shown in Table 2 and Supplementary material exclusion and inclusion criteria). All patients in the observation group were treated upon clinical signs of aGvHD with the first-line standard treatment of aGvHD using corticosteroids and/or second-line treatment, if necessary, at the discretion of the project leaders in each center.

**Table 2:** Incidence of aGvHD, NRM, relapse, and OS in all patients: (a) randomized patients (*N* = 92); (b) patients in the observation group (*N* = 167).

(a) Randomized patients *N* = 92	Placebo patients *N* = 48 (%)	Median days (range)
No aGvHD	24 (50%)	
aGvHD + aGvHD_MS17 + death	24 (50%)	
death_no GvHD (prior day +100)	6	63 (16–82)
aGvHD_MS17 positive prior GvHD signs	18	
aGvHD grade I	10	38 (17–98)
aGvHD grade II	4	38 (19–53)
aGvHD grades III–IV	4	34 (18–50)
NRM	3	194 (128–315)
Relapse	4	227 (141–332)
Overall survival	34	71%

	Prednisolone patients *N* = 44	Median days (range)
No aGvHD	21 (48%)	
aGvHD + aGvHD_MS17 + death	23 (52%)	
death_no GvHD (prior day +100)	1	44
aGvHD_MS17 positive prior GvHD signs	22	
aGvHD grade I	11	44 (17–97)
aGvHD grade II	5	39 (17–61)
aGvHD grades III–IV	6	44.3 (17–74)
NRM	9	174 (109–327)
Relapse	5	161 (104–257)
Overall survival	29	66%
(b) Observation group	*N* = 167 (%)	Median days (range)
aGvHD_MS_17 positive	65 (39%)	
aGvHD I-IV and death	45 (69%)	
death_no GvHD (prior day +100)	4	45 (22–94)
aGvHD_MS17 positive prior GvHD signs	14	
aGvHD grade I	5	53 (12–110)
aGvHD grade II	4	38 (9–47)
aGvHD grades III–IV	5	34 (13–76)
NRM	3	170 (24–351)
Relapse (death)	0	n.a.
Overall survival	11	61%
GvHD signs prior to aGvHD_MS17 CF	26	
aGvHD grade I	17	18 (12–33)
aGvHD grade II	6	25 (13–90)
aGvHD grades III–IV	3	44 (23–74)
NRM	4	146 (88–96)
Relapse (death)	2	418 (351–484)
Overall survival	20	77%
aGvHD_MS17 positive, no GvHD	20	
Septic complications, severe infect	7	
Prednisolone_no GvHD treatment	6	
Randomization problem,consent withdrawal	4	
Renal failure, dialysis	2	
Serum disease	1	
NRM	5	167 (104–318)
Relapse (death)	3	123 (110–134)
Overall survival	12	60%
aGvHD_MS17 negative	102 (61%)	
death_no GvHD (prior day +100)	3	52 (39–72)
aGvHD grade I	29	51 (13–118)
aGvHD grade II	10	38 (18–83)
aGvHD grades III–IV	10	57 (14–92)
NRM	4	111 (39–210)
Relapse	6	231 (111–356)
Overall survival	89	87%

This table summarizes the incidence and severity of acute GvHD (onset: median days from HSCT to aGvHD), NRM, relapse, and overall survival in randomized (a) and observation group (b) patients. Patients in the observation group were subdivided in those with aGvHD_MS17-CF positive samples (*N* = 65) and those who had only negative samples (*N* = 102). For all groups, all grades of aGvHD occurring within 130 days and non-relapse mortality (NRM), death due to relapse, and OS within the 1st year are shown.

### Randomized study population

The intent-to-treat (ITT) population of the randomized phase of the Pre-GvHD trial consisted of 92 patients, 44 receiving prednisolone and 48 receiving placebo for 5 days followed by tapering for 19 days in the absence of clinical signs of aGvHD. One patient in the placebo group was randomized by error despite only negative aGvHD_MS17 test results. In the randomized patients, the median time of aGvHD_MS17 CF sample positivity prior to aGvHD I–IV manifestation was 25 days (mean: 14 days, range: 7–90), in randomization to onset aGvHD: 24 days (0–89). Preemptive therapy was started 2 days after randomization (median 2; range: 0–6 days) in both groups. The as-per-protocol population (PP group; *N* = 84; 42 placebo, 42 prednisolone arm) excluded patients not receiving the study drug for at least 3 days. The data generated in the ITT and the PP groups were similar in all statistical analyses, thus most analysis shown here are on the ITT population. Reasons for premature withdrawal were withdrawal of consent (placebo *N* = 1; prednisolone *N* = 1; observation *N* = 3) and protocol violation (placebo: *N* = 1). One patient in the prednisolone group died prior to randomization (Supplementary Table 2). The reasons for early discontinuation of the study medication in 17 patients (placebo *N* = 11; prednisolone *N* = 6) are summarized in Supplementary Table 3. Unblinding due to signs of clinical manifest aGvHD II or higher occurred in 14 patients (placebo *N* = 9; prednisolone *N* = 5).

### Outcome of the randomized phase of the PRE-GVHD trial

**Fig. 2:**
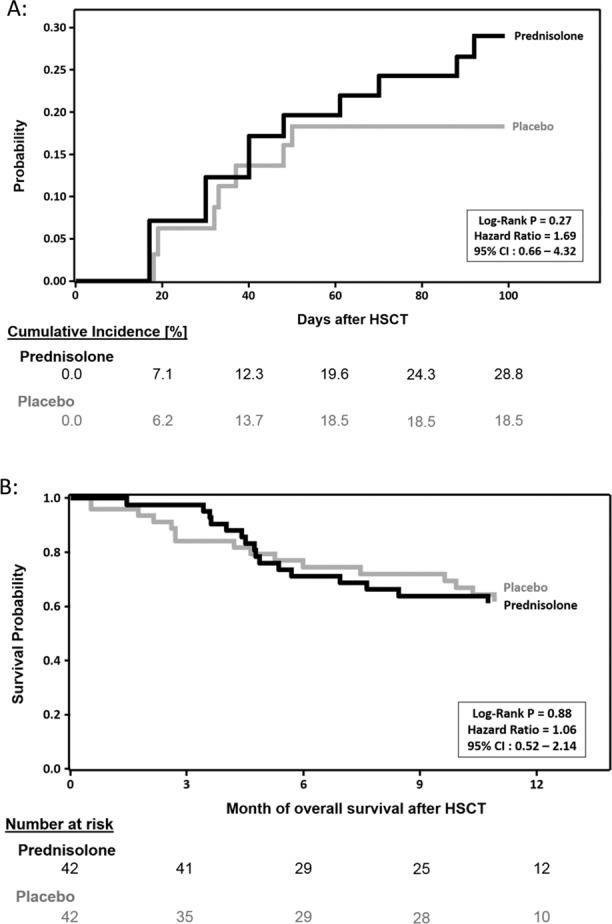
Outcome of the Pre-GvHD trial. **A** Cumulative incidence of acute GvHD (aGvHD) grades II–IV up to day +100 in the ITT population of randomized patients receiving prednisolone (*N* = 44, black solid line) or placebo (*N* = 48, gray solid line), with death considered as competing event. Time to aGvHD grade II or higher or death is left truncated at the time of randomization. aGvHD grade ≥ II until day +100 occurred in 12 patients of the prednisolone and in eight of the placebo group. Death by day +100 occurred in one patient in the prednisolone and four patients in the placebo group. **B** Overall survival probability in the prednisolone (*N* = 44, black solid line) and placebo (*N* = 48, gray solid line) ITT population during follow-up of 1 year after HSCT. Overall survival time is left truncated at the time of randomization.

**Fig. 3:**
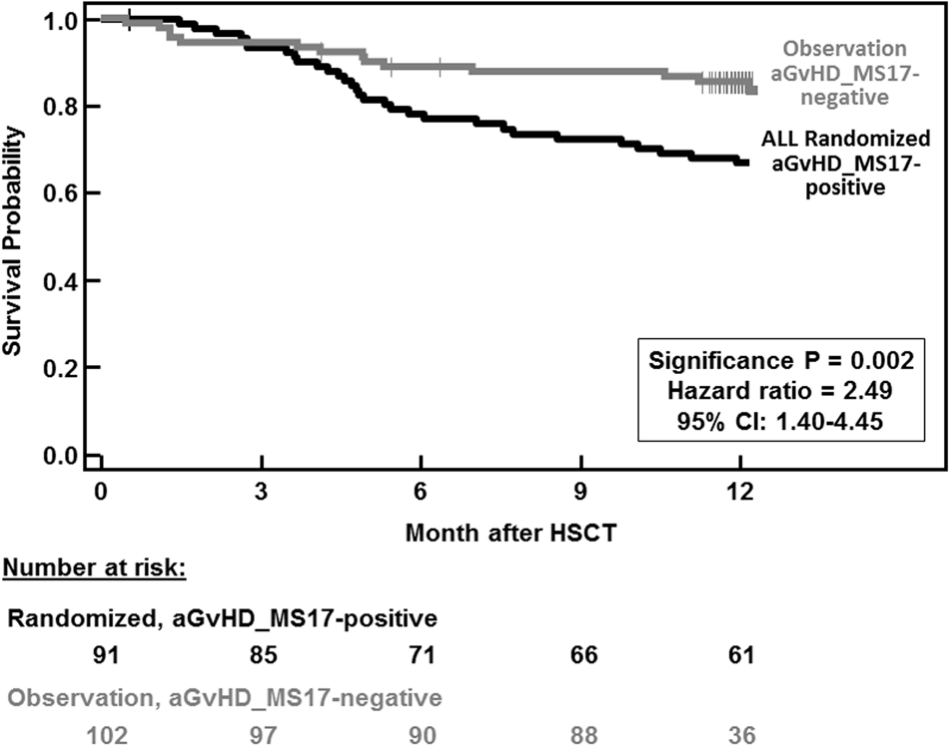
Overall survival probability in the randomized versus the observation group patients. One year OS probability after HSCT is shown for all randomized (*N* = 91, black solid line), aGvHD_MS17 pattern positive patients (*N* = 91; CF > 0.1) and is compared to those of the observation group patients (*N* = 102, gray solid line) with only negative samples for the AGvHD_MS17 CF (CF < 0.1).

The primary endpoint was the incidence of aGvHD II–IV between randomization and day +100 after HSCT. The incidence of aGvHD grades II–IV was 25% in the randomized patients. Death during the first 100 days was considered an event also in patients without aGvHD II–IV. Figure 2A shows that the cumulative incidence of aGvHD II–IV, with death as competing event, was similar in the prednisolone and placebo groups (HR: 1.69, 95% CI: 0.66–4.32, *P* = 0.27). Table 2 summarizes the incidence and severity of aGvHD, NRM, and OS in the placebo, prednisolone (Table 2a), and the observation groups (Table 2b). Figure 2B shows the OS in the PP group. The probability of OS was similar in both arms (HR: 1.06; 95% CI: 0.52–2.14; *P* = 0.88; Supplementary Table 4) as it was in the ITT population. Within 100 days after HSCT, four patients in the placebo and one patient with aGvHD grade II in the prednisolone group died, but by day 130 this slight difference was lost (seven and five deaths, respectively). In the 1-year follow-up, 15 and 16 patients died, respectively (Supplementary Table 4). The main causes of death were relapse (placebo: 6/48; 13%; prednisolone: 5/44; 11%) followed by NRM (placebo: 4/48; 8%; prednisolone: 4/44; 9%) and other causes (placebo: 4/48; prednisolone: 7/44; summarized in Supplementary Table 5). The difference in time from HSCT to NRM was not analyzed, due to the low number of events in both randomized treatment arms. Patients with only aGvHD_MS17 CF negative test results in the observation group (*N* = 102) had a very low risk to die compared to the randomized patients with aGvHD_MS17 positive samples (*N* = 91), who had a 2.5-fold (*P* = 0.002) increased risk of death within 1 year post HSCT (Fig. 3). The cumulative incidence of leukemic relapse or progression (including death due to relapse/progression) at 1 year after HSCT was slightly higher in the placebo group (20%) than in the prednisolone group (14%), but the difference was not statistically significant (*P* = 0.46). In this analysis, death unrelated to relapse or progression was considered as competing risk and patients lost to follow-up were considered as censored cases (Supplementary Table 6). The frequency of infections during the screening period prior to randomization was already higher in the prednisolone group (23%) than in the placebo group (8%). After the screening period, infections were reported in 91% of the patients in the prednisolone group and in 83% of the patients in the placebo group (Supplementary Table 7). The slightly higher frequency of infections was observed at all time points during the study, with the exception of the final visit at 1 year after allo-HSCT.

### Safety data

The mean duration of treatment (including taper) was 16 days in the placebo group and 18 days in the prednisolone group (Supplementary Table 8). The shorter treatment duration in the placebo arm is explained by the higher frequency of premature discontinuation of the study medication in the placebo group (27%) compared to 17% in the prednisolone group. AEs observed in the two treatment groups are summarized in Supplementary Table 8. The frequency of AEs was higher in the placebo when compared to the prednisolone group (64% and 50%, respectively). The most common AEs were diarrhea (18% and 10%, respectively), nausea (11 and 5%), vomiting (4 and 5%), and acute kidney injury (7 against 2%). Serious AEs were more frequent in the placebo group (13 and 7%). The frequency of (S)AEs attributed to the study medication by the investigators was lower in the placebo (13%) than in the prednisolone group (21%). AEs leading to withdrawal from study drug were only observed in the placebo group (five patients with gastrointestinal complications, one GvHD of the skin, one patient with insomnia, and one with acute kidney injury). In addition, one placebo patient died from pulmonary hemorrhage (Supplementary Table 8).

## Discussion

This is the first prospective randomized multicenter trial using proteomic peptide profiling of patients to assess the effect of preemptive GvHD-directed therapy post-HSCT. With sufficient numbers of patients randomized and long follow-up, the results are disillusioning and reflect the outcome of previous attempts of preemptive therapy. The preemptive treatment of imminent aGvHD with 2–2.5 mg prednisolone/kg BW for 5 consecutive days after randomization did not reduce the clinical development of severe aGvHD. Other drugs, like etanercept or ruxolitinib, may be more effective than corticosteroids, but this would have to be tested in another clinical trial. Several reasons may account for this negative outcome. The short treatment period of only 5 days followed by tapering for 19 days (mean treatment time: 16 days placebo; 18 days prednisolone; Supplementary Table 3) may not be sufficient to inhibit the proliferation of allogeneic T cells that induce aGvHD. Lower overall aGvHD rates in the current trial could be due to differences in the patient population as compared to our previous studies [11, 13]. The pilot study leading to the design of the present trial (Supplementary Table 9) had more patients with no CR and included mismatch transplantation. In the Pre-GvHD trial, only good-risk patients in CR, chronic phase of chronic myeloid leukemia, very good PR, or patients with untreated MDS were included until 2013. Only when recruitment was still not completed by 2013, the inclusion criteria were expanded to include patients transplanted in relapse or refractory patients with more than 10% leukemic blasts (AML, ALL, and MDS/MPN). In the previous pilot, the majority of the patients were transplanted after relapse or treatment failure. Furthermore, although conditioning regimens did not influence the data significantly in former analyses, some RIC protocols are designed to allow for more allogeneic reactions. For example, in the previous analyses the intensive fludarabine–cytosine arabinoside–amsacrine (FLAMSA) [25] either with TBI or busulfan and ATG protocol was the most commonly applied RIC, while in the current trial protocols without particular influence on immune reactions such as fludarabine/melphalan or fludarabine/busulfan made up more than 50% of the RIC protocols (Supplementary Table 11). All these factors together may explain the lower aGvHD rate in the Pre-GvHD trial compared to previous studies. Considering safety of preemptive therapy with prednisolone, neither infections including virus reactivations (Supplementary Table 7), nor disease relapses (Supplementary Table 6) differed significantly between the prednisolone and the placebo arms. In addition, the frequency of other AEs was not increased in the prednisolone group when compared to the placebo group (Supplementary Table 8). Thus, no specific safety risk of the preemptive therapy with prednisolone was identified, although this was a relatively small patient number to detect rare events.

There was a slight difference in aGvHD development in the randomized patient groups. As shown in Table 2, the incidence of aGvHD grades II–IV was slightly higher in the prednisolone group (*N* = 11) compared to the placebo group (*N* = 8). This may be explained by more HLA-mismatched donors (*N* = 5 (10%) placebo; *N* = 8 (18%) prednisolone) and higher rate of gender mismatch HSCT in the prednisolone group (male recipients transplanted from female donors in the placebo *N* = 6 (12%); prednisolone *N* = 9 (20%); Table 1).

We can draw further conclusions from the follow-up of the patients who entered this large multicenter trial but were not randomized. aGvHD_MS17 CF positivity predicted NRM and lower OS within the 1st year after HSCT. Patients with only aGvHD_MS17 CF negative samples (observation group; *N* = 102) had a lower risk for NRM compared to patients with at least one aGvHD_MS17-positive test (randomized patients; *N* = 91; HR: 2.49; *P* = 0.002; Fig. 3). Preemptive therapy based on laboratory biomarkers was investigated by Bacigalupo et al. [17], using ATG treatment in patients after HSCT. They found that the highest risk group benefitted from this treatment. Later a prospective randomized trial of prednisolone treatment (1 mg/kg) or placebo included patients with aGvHD grade I. The outcome was that aGvHD grade II development was reduced, but infectious complications were higher and the incidence of aGvHD III–IV was not reduced [18]. In our trial, the incidence of infectious complications post preemptive prednisolone treatment was not significantly different to those in the placebo group. The most promising plasma diagnostic biomarkers appear to be regenerating islet-derived protein 3 alpha (Reg-3a; [26]), suppression of tumorigenicity 2 (ST2; [27]), and soluble TNF-receptor 1 (sTNFR1; [28]) as diagnostic markers for GvHD and prediction of performance after HSCT. Reg-3a was tested in samples from 1014 HSCT patients from three transplantation centers [29]. Recently, the so-called Ann Arbor Score [15, 28, 30] of aGvHD, which relies on three biomarkers (Reg-3a, ST2, and sTNFR1), was implemented in the analysis of patients after HSCT. It predicts treatment response by day 28 post GvHD therapy and 6-month NRM irrespective of center-specific strategies. The international Mount Sinai Acute GvHD International Consortium has been recently founded and a prospective monitoring study for correct diagnosis and NRM prediction is currently ongoing in an international setting. Our findings add to the current literature where a cluster of plasma proteins detected by ELISA were used to predict outcome after HSCT. Two to six differentially secreted plasma biomarkers after clinical diagnosis of aGvHD grade II or higher indicated decreased OS and increased NRM [14–16]. Prediction of OS and NRM was also studied by Luft et al. [31], who used an “Endothelial Activation and Stress Index” (EASIX, an algorithm using lactate dehydrogenase (U/L) multiplied by creatinine (mg/dL) and divided by platelets (10^9^ cells per L)) to predict NRM and death in patients after HSCT [31, 32]. However, only aGvHD_MS17 can be used to predict overall aGvHD and/or to guide preemptive therapy in patients after HSCT without prior clinical aGvHD diagnosis. In addition, aGvHD_MS17 monitoring has the potential to characterize new biomarkers/pathways involved in the development of aGvHD and may be useful to detect new alternative and much needed therapeutic targets for treatment of severe aGvHD. In addition, combinations with the other biomarkers, like the EASIX score, used for predicting outcome prior to HSCT [23] may be used to pre-select high-risk patients for further analysis with aGvHD_MS17. Application of aGvHD_MS17 monitoring between days +7 and +21 led to a higher predictive value of aGvHD than at later time points. An early analysis (e.g., day +14 (±7) post HSCT) using aGvHD_MS17 in the clinical practice could be helpful to recognize patients at high risk to develop aGvHD and/or is prone to NRM within the 1st year after HSCT. In our study, we observed that the previously established aGvHD_MS17 urine peptide marker pattern allowed the prediction of 2.5-fold higher NRM. Thus, a positive aGvHD_MS17 test result by day +14 may help to guide intensified clinical monitoring of patients with high risk of developing aGvHD or NRM to administer effective other immunosuppressive therapy or intensified care early on.

## Supplementary information

suplemental material
