# Supplementary material for: A multicenter prospective, randomized, placebo-controlled phase II/III trial for preemptive acute graft-versus-host disease therapy
Source: Leukemia. 2020 Oct 20;35(6):1763–72. doi: 10.1038/s41375-020-01059-3 (PMC8179847; doi:10.1038/s41375-020-01059-3)
Supplement: Supplementary file 1 — suplemental material [file 41375_2020_1059_MOESM1_ESM.docx]

Supplemental Material:

Inclusion Criteria:

- Written informed consent
- All patients ≥ 18 years on day +7 (±3) after 1^st^ allo-HSCT
- Patients transplanted for acute myeloid or lymphoid leukemia in CR (<5% leukemic blast cells in the bone marrow) or PR (<20% leukemic blast cells in the bone marrow); myelodysplastic and/or myeloproliferative syndromes: untreated, CR, PR; lymphomas: PR; chronic myeloid leukemia: CP; chronic lymphatic leukemia: CR, PR; multiple myeloma: CR, PR; severe aplastic anemia at the time of allogeneic HSCT
- aGvHD prophylaxis with any combination of the following: e.g. cyclosporin A (CsA), methotrexate (MTX), mycophenolic acid (MMF), sirolimus, tacrolimus, everolimus and others with or without immunosuppressive antibodies (e.g. antithymocyte globulin (ATG))
- Woman of childbearing potential must have a negative pregnancy test prior to HSCT
- Sufficient contraceptive methods for men and women of reproductive age at the beginning of the study and during the study and consent of the patient to use a sufficient contraceptive method within 6 months after the end of the study. Permitted double contraceptive methods are implants, injection preparation, intrauterine devices, portal cap, sterilization, hysterectomy, condoms, spermicide, vasectomy and/or sexual abstinence.

Prior to randomization:

• Positivity of aGvHD-specific proteomic pattern

Exclusion Criteria

- patients after ≥ 2^nd^ allo-HSCT
- patients transplanted in relapse of their underlying disease (AML/ALL: ≥ 20 % leukemic blast cells in the bone marrow)
- transplantation with CD34+ enriched- or ex vivo T-cell-depleted stem cells, transplantation from syngeneic or haploidentical or cord blood donors
- steroids as part of the aGvHD prophylaxis
- pregnant or nursing women
- participation in another therapeutic study within 30 days before and during this study

Prior to randomization:

- patients with acute GvHD grade II to IV
- acute renal failure (≥ 2x upper normal boundary of serum creatinine)
- serious life-threatening infection at the time of sampling for acute GvHD proteomic pattern
- relapse or progression of underlying disease

Patients enrolled, who cannot be randomized, will be followed-up in the observational group

**Reasons for no Randomization of patients with aGvHD_MS17 positive samples:**

**Methods**:

**Sample preparation, CE-MS analysis and data analysis**:

Briefly, urine samples were thawed and the pH is adjusted to pH 10 using ammonia and cleared by centrifugation for 10 min at 13,000×g at 4°C prior to CE-MS analysis. The supernatants were filtered using Pharmacia C2-column (Amersham Bioscience, Buckinghamshire, UK). Polypeptides are eluted with 50% (v/v) acetonitrile (Sigma-Aldrich, Taufkirchen, Germany) in 20 μl HPLC-grade water (Roth, Karlsruhe, Germany) containing 0.5% (v/v) formic acid (Sigma-Aldrich). After lyophilization, and the pellet is suspended in 20 μl HPLC-grade water, sonicated for 1 min in an ultrasonic bath, centrifuged for 10 min at 13,000×g, and injected into the CE.

**CE-MS analysis** by on-line coupling of a P/ACE-MDQ capillary electrophoresis unit (Beckman Coulter, Fullerton, USA) to a Micro-TOF mass spectrometer (Bruker Daltronic, Bremen, Germany) was performed as previously described. ^1,2^ Quality control criteria for an adequate CE-MS peptide profile were: detection of more than 300 annotated peptides with a minimal mass resolution of 8,000 and migration time interval of 10 min.

The application of a software (MosaVisu) allows visualization of the CE-MS data and generation of aGvHD-specific polypeptide profiles is achieved by application of an additional software (MosaCluster) based on support vector machines (SVM). ^3, 4^ The aGvHD_MS17 file consists of 17 differentially excreted peptides, like specific fragments of collagen-alpha-1 and alpha-2, fragments of beta-2 microglobulin, the CD99 antigen, fibronectin precursor and a N-terminal fragment of serum albumin. MosaCluster lead to a classification factor (CF), a dimensionless number based on the 17 differentially excreted peptides and a CF of greater than 0.1 was established for prediction of aGvHD.

| **Criteria for evaluation:**  Efficacy:  The primary endpoint was defined as the occurrence of aGvHD ≥grade II between time of randomization and  100 days after HSCT. If a death occurs between randomization and 100 days after HSCT in a patient without  aGvHD (≥ grade II), then this was also considered as treatment failure, equivalent to an aGvHD (≥ grade II).  The secondary efficacy endpoints are   1. the severity of aGvHD between time of randomization and 100 days after allo-HSCT, 2. the occurrence of aGvHD (≥ grade II) between time of randomization and end of follow-up for aGvHD (i.e. 130 days after allo-HSCT) 3. the severity of aGvHD between time of randomization and end of follow-up for aGvHD (i.e. 130 days after allo-HSCT) 4. overall survival 5. transplant-related mortality (TRM) 6. the occurrence of leukemic relapses and progression 7. the occurrence of infections.   Safety:  The exposure of study treatment was characterized by number of administrations and the cumulative dose for both treatment groups.  AEs were coded using the Medical Dictionary for Regulatory Activities (MedDRA), version 19.1.  Annual Safety Reports were written and submitted to National Competent Authority and leading Ethic committee as required by ICH guidelines and national regulations. |
| --- |

| **Statistical methods:**  Sample Size Calculation:  In total 78 evaluable patients (2 x 39) were required to detect a reduction in the incidence of aGvHD (grade II-IV) or death from 80% in the placebo group to 50% (odds ratio =0.3) in the active treatment group with a type I error of 5% and a power of 80% using a two-sided Cochran-Mantel-Haenszel test. The number of patients to be randomized was increased to 2 x 45 patients to take into account a reduced effect caused by protocol deviations or other confounding factors.  Analysis populations:  The primary analysis population is intent-to-treat (ITT) population, which is defined as all patients randomized. Patients in the ITT population were analyzed as randomized. In addition, all relevant efficacy endpoints were also analyzed for the per protocol population. The per-protocol population included all randomized patients, who fulfilled the key inclusion criteria and received double-blind treatment for at least 3 days (pre-emptive dose of ≥ 2 mg/kg). Patients in the per-protocol population were analyzed as treated.  The safety population included all patients who were randomized and received double-blind study medication. Patients in the safety population were analyzed as treated.  Patients who were not randomized were included into the observational study group.  Analysis of primary endpoint:  The two-sided Cochran-Mantel-Haenszel (CMH) test was used for the analysis of the primary endpoint. Odds-ratio and relative risk for developing aGvHD (grade II-IV) in the two treatment arms and corresponding 95% confidence intervals were calculated. In addition to the unstratified analysis also an analysis stratified by study site was performed (the study site was used as stratification factor for randomization).  Analysis of other study endpoints: The following categorical secondary endpoints were analyzed using the two-sided Cochran-Mantel-Haenszel test for 2 x r frequency tables:   - Severity of aGvHD between randomization and 100 days after allo-HSCT using the following grading:   1. no aGvHD, grade I, grade II, grade III, grade IV.   2. no aGvHD, grade I, grade II, grade III, grade IV, grade V. Grade V was assigned if the patient died between time of randomization and 100 days after allo-HSCT. - Occurrence of all aGvHD ≥ grade II with onset between randomization and the visit used to record late onset aGvHD (i.e. study day 130). In agreement with the primary endpoint, deaths in patients without an aGvHD (grade II) between randomization and 130 days after HSCT will be considered as treatment failure, equivalent to an aGvHD ≥ grade II). - Severity of all aGvHD after randomization until 130 days after allo-HSCT using the following grading:   1. no aGvHD, grade I, grade II, grade III, grade IV.   2. no aGvHD, grade I, grade II, grade III, grade IV, grade V. Grade V was assigned if the patient died between time of randomization and 130 days after allo-HSCT.   For each patient the most severe grade of an aGvHD was used in the analyses of severity.  The endpoint overall survival time was defined as time from HSCT to death for any cause within the first year after HSCT. The overall survival time from HSCT until death was left-truncated for all randomized patients, because no death can occur prior to randomization. The overall survival time was right censored in patients, who were lost-to-follow-up prior to 1 year post-HSCT or were still alive at 1 year post-HSCT. Overall survival time were analyzed using Cox proportional hazard models considering left truncation at time of randomization and right censoring as described above. The overall survival rate for selected time points (3, 6, 9 and 12 months after allo-HSCT) for the two groups were determined together with the hazard ratio (HR), the 95% confidence intervals for the HR, and the p-value of corresponding Wald chi-square test.  The cause of death were summarized in a frequency table included the frequency of transplant-related death.  The time until occurrence of leukemic progression or relapse (=event) was analyzed using competing risk method, i.e. death due to a reason not related to leukemic relapse or progression were considered as a competing risk in the analysis and patient know to be alive at 1 year post-HSCT or at the last date prior to lost-to-follow-up were considered as censored events. Left-truncation of the time to leukemic progression was considered consistent with the OS analysis. Cumulative incidence rates were calculated for leukemic progression or relapse at selected time points and the two groups were compared using the Wald test included estimates of the hazard ration and corresponding 95% CIs.  All statistical tests regarding the secondary endpoints at a two-sided alpha-level of 0.05 were of exploratory nature, and performed to support the findings for the primary endpoints. For this reason no alpha-adjustment were applied for multiple testing.  Descriptive statistical methods were used to compare the safety data of the two randomized treatment groups and for all data collected in the observational group.  For any AEs, related AEs, serious AEs, related serious AEs, AEs leading to death and AEs leading to premature discontinuation of study medication separate frequency tables were generated presenting the AEs by MedDRA preferred terms, system organ classes (SOC) and treatment groups. In addition to the frequency of death the cause of death were summarized for the two treatment groups and the observational group. |
| --- |

| Suppl Table 1 Disposition of Patients  Analysis: All Patients Enrolled    ­­­­­­­­­­­­­­­­­­­­­­­­­­­­­­­­­­­­­­­­­­­­­­­­­­­­­­­­­­­­­­­­­­­­­­­­­­­­­­­­­­­­­­­­­­­­­­­­­­­­­­­­­­­­­­­­­    Patients enrolled (informed consent) 267 (100.0%)    Patients eligible for study (all inclusion criteria fulfilled, no exclusion criteria met) 263 ( 98.5%)    Patients not eligible for study, but randomized 1 ( 0.4%)    Patients in the ITT population (Randomized and Observational) 259 ( 97.0%)  - Placebo 48 ( 18.0%)  - Prednisolone 44 ( 16.5%)  - Observational 167 ( 62.5%)    Patients in the safety population (Randomized) 87 ( 32.6%)  - Placebo 45 ( 16.9%)  - Prednisolone 42 ( 15.7%)    Patients in the per-protocol population (Randomized) 84 ( 31.5%)  - Placebo 42 ( 15.7%)  - Prednisolone 42 ( 15.7%)    Patients in the ITT population who started pre-emptive therapy 87 ( 32.6%)  - Placebo 45 ( 16.9%)  - Prednisolone 42 ( 15.7%)    Patients who discontinued pre-emptive therapy prematurely 19 ( 7.1%)  - Placebo 12 ( 4.5%)  - Prednisolone 7 ( 2.6%)  ------------------------------------------------------------------------------------------------------------------  Percentages are based on number of patients enrolled.    **Suppl. Table 1: Summary of Patient Disposition**  Of the 267 patients enrolled 92 patients were randomized to one of the two treatment groups (Placebo: n=48, Prednisolone: n=44), and 167 patients entered the observational group (see Table 1). The reasons are summarized in Table 2.  Suppl Table 2 Summary of Reasons for Premature Withdrawal from the Study  Analysis: Intent-to-Treat Population (plus Observational)  ­­­­­­­­­­­­­­­­­­­­­­­­­­­­­­­­­­­­­­­­­­­­­­­­­­­­-­­­­­­­­­­­­­­­­­­­­­­­­­­­­­­­­­­­­­­­­­­­­­­­­­­­­­    Placebo Prednisolone All Randomized Observation    (N = 48) (N = 44) (N = 92) (N = 167)  ----------------------------------------------------------------------------------------------------------    Protocol violation   1 ( 2.1%)   0 ( 0.0%)   1 ( 1.1%)   0 ( 0.0%)  Consent withdrawn   1 ( 2.1%)   1 ( 2.3%)   2 ( 2.2%)   3 ( 1.8%)  Lost to follow-up   0 ( 0.0%)   1 ( 2.3%)   1 ( 1.1%)   0 ( 0.0%)  Other reason   1 ( 2.1%)   0 ( 0.0%)   1 ( 1.1%)   0 ( 0.0%)  Total   3 ( 6.3%)   2 ( 4.5%)   5 ( 5.4%)   3 ( 1.8%)  ----------------------------------------------------------------------------------------------------------  Percentages are based on N.  *Patients who died are considered to have completed the study.  _______________________________________________________________________________________________________________________________________________  **Suppl Table 3: Summary of discontinuation of study medication**  Analysis: Safety Population  ­­­­­­­­­­­­­­­­­­­­­­­­­­­­­­­­­­­­­­­­­­­­­­­­­­­­­­­­­­­­­­­­­­­­­­­­­­­­­­­­­­­­­­­­­­­­­­------------  Placebo Prednisolone Total  (N=45) (N=42) (N=87)  ­­­­­­­­­­­­­­­­­­­­­­­­­­­­­­­­­­­­­­­­­­­­­­­­­­­­­­­­­­­­­­­­­­­­­­­­­­­­­­­­­­­­­­­­­­­­­­    Unblinded  Yes 11 ( 24.4%) 6 ( 14.3%) 17 ( 19.5%)  No 34 ( 75.6%) 36 ( 85.7%) 70 ( 80.5%)  Total (N) 45 42 87  ­­­­­­­­­­­­­­­­­­­­­­­­­­­­­­­­­­­­­­­­­­­­­­­­­­­­­­­­­­­­­­­­­­­­­­­­­­­­­­­­­­­­­­­­­­­­­­  Reason for unblinding  Acute GvHD Grade II – IV 9 ( 20.0%) 5 ( 11.9%) 14 ( 16.1%)  Other reason 2 ( 4.4%) 1 ( 2.4%) 3 ( 3.4%)  Total (N) 45 42 87    ­­­­­­­­­­­­­­­­­­­­­­­­­­­­­­­­­­­­­­­­­­­­­­­­­­­­­­­­­­­­­­­­­­­­­­­­­­­­­­­­­­­­­­­­­­­­­­------------  **Suppl Table 3 continued: Summary of discontinuation of study medication**  ­­­­­­­­­­­­­­­­­­­­­­­­­­­­­­­­­­­­­­­­­­­­­­­­­­­­­­­­­­­­­­­­­­­­­­­­­­­­­­­­­­­­­­­­­­­­­­------------  Placebo Prednisolone Total  (N=45) (N=42) (N=87)  ­­­­­­­­­­­­­­­­­­­­­­­­­­­­­­­­­­­­­­­­­­­­­­­­­­­­­­­­­­­­­­­­­­­­­­­­­­­­­­­­­­­­­­­­­­­­­­-----------------------------------------------------------------------------  Study treatment discontinued prematurely  Yes 12 ( 26.7%) 7 ( 16.7%) 19 ( 21.8%)  No 33 ( 73.3%) 35 ( 83.3%) 68 ( 78.2%)  n 45 42 87  ­­­­­­­­­­­­­­­­­­­­­­­­­­­­­­­­­­­­­­­­­­­­­­­­­­­­­­­­­­­­­­­­­­­­­­­­­­­­­­­­­­­­­­­­­­­­­­----------  Main reason for study treatment discontinuation  Acute GvHD Grade II – IV 8 ( 17.8%) 4 ( 9.5%) 12 ( 13.8%)  Life-threatening infection(s) 1 ( 2.2%) 0 1 ( 1.1%)  Other reason 3 ( 6.7%) 3 ( 7.1%) 6 ( 6.9%)  n 45 42 87  _______________________________________________________________________________________________________________________________________________  Suppl Table 4 Overall Survival Time from allo-HSCT for one year  Analysis: Intent-to-Treat Population  ­­­­­­­­­­­­­­­­­­­­­­­­­­­­­­­­­­­­­­­­­­­­­­­­­­­--­­­­­­­­­­­­­­­­­­­­­­­­­­­­­­­­­­­­­­­­­­­­­­­­­­­  Placebo Prednisolone  N=48 N=44  ­­­­­­­­­­­­­----­­­­­­­­­­­­­­­­­­­­­­­­­­­­­----­­­­­­­­­­­­­­­­­­­­­­­­­­­­­­­­­----­­­­­­­­­­­­­­­­­  Number of patients died 15   16  ­­­­­­­­­­­­­­­­­­­­­­­­­­­­­­­­­­­­­­­­­­­­­­­­­­­­­­­­­­­­­­­­­­­­­­­­­­­­­­­­­­­­­­­­­­­­­­­­­­­­­­­­  No. of pat. Survival Estimates No. of pat. Survival Estimates  failed/at [95% CI] failed/at [95% CI]  Survival estimated (§) at risk(+) risk(+)  3 months 6/ 39 85.6% [ 75.4%, 97.2%] 1/ 42 97.5% [ 92.9%, 100.0%]  6 months 10/ 33 76.8% [ 65.1%, 90.6%] 12/ 30 71.9% [ 59.6%, 86.7%]  9 months 11/ 32 74.5% [ 62.5%, 88.8%] 15/ 26 64.7% [ 51.8%, 80.8%]  12 months 15/ 11 65.3% [ 52.4%, 81.2%] 16/ 12 62.2% [ 49.1%, 78.7%]  --------------------------------------------------------------------------------------------------------  HR(#) [95% CI] 1.15 [ 0.57, 2.33]  Wald chi-square test P=0.6951(*)  --------------------------------------------------------------------------------------------------------  (#) Hazard ratio and corresponding 95% confidence interval based on Cox proportional hazard model:  HR = hazard rate prednisolone divided by hazard rate placebo  (§) Overall survival is left truncated at time of randomization  (*) Two-sided Wald chi-square test (p-value) for difference between the two survival curves  (+) Number of patients failed = Number of patients who died; Number of patients left = Number of remaining patients under risk    Suppl Table 5 Summary of Cause of Death (first year post-HSCT)  Analysis: Intent-to-Treat Population (plus Observational) Percentages are based on N (number of patients in treatment group).  ­­­­­­­­­­­­­­­­­­­­­­­­­­­­­­­­­­­­­­­­­­­­­­­­­­­­­­­­­­­­­­­­­­­­­­­­­­­­­­­­­­­­­­­­­­­­­­­­­­­­­­­­­­­­­­­­­­­­­­­­­­­­­­­­­  Placebo Prednisolone Observation  (N=48) (N=44) ( (N=167)  ­­­­­­­­­­­­­­­­­­­­­­­­­­­­­­­­­­­­­­­­­­­­­­­­­­­­­­­­­­­­­­­­­­­­­­­­­­­­­­­­­­­­­­­­­­­­­­­­­­­­­­­­­­­­­­----­­­­­­­­­­­­­­­  Total number of patients died 15 ( 31.3%) 16 ( 36.4%) 34 ( 20.4%)  Relapse or progression 6 ( 12.5%) 5 ( 11.4%) 11 ( 6.6%)  Secondary malignancy 1 ( 2.1%) 0 0  **Transplant related** (GvHD) 4 ( 8.3%) 4 ( 9.1%) 12 ( 7.2%)  Acute GvHD 1 ( 2.1%) 2 ( 4.5%) 2 ( 1.2%)  Chronic GvHD 0 0 1 ( 0.6%)  Infection and GvHD 3 ( 6.3%) 2 ( 4.5%) 6 ( 3.6%)  **Transplant related** (not GvHD)  Viral Encephalitis 0 0 1 ( 0.6%)  Lung AEs 4 ( 8.3%) 7 ( 15.9%) 11 ( 6.6%)  Interstitial pneumonia 0 0 3 ( 1.8%)  Atypical pneumonia 0 1 ( 2.3%) 0  Idiopathic pneumonia, respiratory Insufficiency 0 0 1 ( 0.6%)  Nosocomial pneumonia 0 0 1 ( 0.6%)  Pulmonary infection with Pseudomonas aeruginosa 0 1 ( 2.3%) 0  Respiratory failure, atypical pneumonia 1 ( 2.1%) 0 1 ( 0.6%)  Pneumonia 0 1 ( 2.3%) 0  Respiratory insufficiency pneumonia 0 0  Heart AEs  Chronic cardiac failure (chronic) 0 1 ( 2.3%) 0  Ventricular fibrillation 0 0 1 ( 0.6%)  Coma, hypoxia, organ failure 0 0 1  Septic complications  Multi organ failure (MOV) 1 ( 2.1%) 0 1 (0.6%)  Organ failure by septic shock 0 0 1 ( 0.6%)  Sepsis 0 0 1 ( 0.6%)  Sepsis and pneumonia 0 0 1 ( 0.6%)  Sepsis, MOV 0 0 1 ( 0.6%)  Septic shock 1 ( 2.1%) 1 ( 2.3%) 0  Septic shock with MOV 0 1 ( 2.3%) 0  Status epilepticus, Sepsis, MOV 1 ( 2.1%) 0 0  Unknown 0 1 ( 2.3%) 0        Suppl Table 6 Cumulative Incidence of Leukemic Relapse or Progression  Analysis: Intent-to-Treat Population  ­­­­­­­­­­­­­­­­­­­­­­­­­­­­­­­­­­­­­­­­­­­­­­­­­­­­­­­­­­--­­­­­­­­­­­­­­­­­­­­­­­­­­­­­­­­­­­­­­­­­­­­­­­­­­­­­­­­­­  Placebo Prednisolone  N=48 N=44  ­­­­­­­­­­­­­­­­­­­­­­­­­­­­­­­­­­­­­­­­­­­­­­­­­­­­­­­­­­­­­­­­­­­­­­­­­­­­­­­­­­­­­­­­­­­­­­­­­­­­­­­­­­­­­­­­­­­­­­    Number of patients with 9 6  leukemic relapse or  progression  ----------------------------------------------------------------------------------------------------------------------  Number of patients with 9 11  death unrelated to relapse  or progression  (competing risk)  ----------------------------------------------------------------------------------------------------------------------  Number of patients censored 30 27  ----------------------------------------------------------------------------------------------------------------------  Cumulative relapse/ Cumulative 95% Confidence Interval Cumulative 95% Confidence Interval  progression rate (§) Incidence (%) Incidence (%)  allo-HSCT 0 - 0 -  3 months 8.8% [ 3.5%, 21.8%] 2.4% [ 0.4%, 13.3%]  6 months 15.4% [ 8.0%, 29.7%] 14.0% [ 6.4%, 30.6%]  9 months 17.7% [ 9.2%, 34.1%] 14.0% [ 6.4%, 30.6%]  12 months 20.2% [ 10.8%, 37.5%] 14.0% [ 6.4%, 30.6%]  ----------------------------------------------------------------------------------------------------------------------  HR(#) [95% CI] 0.69 [ 0.25, 1.90]  Wald chi-square test P=0.4678(*)  ----------------------------------------------------------------------------------------------------------------------  (#) Hazard ratio and corresponding 95% confidence interval based on Cox proportional hazard model:  HR = hazard rate prednisolone divided by hazard rate placebo  (§) Time to relapse or progression is left truncated at time of randomization    **Suppl Table 7 Summary of Infections Over Time**  Analysis: Intent-to-Treat Population (plus Observational)  ­­­­­­­­­­­­­­­­­­­­­­­­­­­­­­­­­­­­­­­­­­­­­­­­­­­­­­­­­-­­­­­­­­­­­­­­­­­­­­­­­­­­­­­­­­­­­­­­­­­­­­­­­­­­­­­­­­­    Time point Infections Placebo Prednisolone All Randomized Observational  -------------------------------------------------------------------------------------------------------------------    Screening Yes   4 ( 8.3%)   10 ( 22.7%)   14 ( 15.2%)   25 ( 15.0%)  No   44 ( 91.7%)   34 ( 77.3%)   78 ( 84.8%)   142 ( 85.0%)  Total   48 (100.0%)   44 (100.0%)   92 (100.0%)   167 (100.0%)    At any visit post-screening Yes   40 ( 83.3%)   40 ( 90.9%)   80 ( 87.0%)   128 ( 76.6%)  No   8 ( 16.7%)   4 ( 9.1%)   12 ( 13.0%)   39 ( 23.4%)  Total   48 (100.0%)   44 (100.0%)   92 (100.0%)   167 (100.0%)    At any visit post-randomization Yes   38 ( 84.4%)   38 ( 90.5%)   76 ( 87.4%)   0 ( 0.0%)  No   7 ( 15.6%)   4 ( 9.5%)   11 ( 12.6%)   0 ( 0.0%)  Total   45 (100.0%)   42 (100.0%)   87 (100.0%)   0 ( 0.0%)    Day  14 Yes   13 ( 27.1%)   16 ( 37.2%)   29 ( 31.9%)   58 ( 35.4%)  No   35 ( 72.9%)   27 ( 62.8%)   62 ( 68.1%)   106 ( 64.6%)  Total   48 (100.0%)   43 (100.0%)   91 (100.0%)   164 (100.0%)    Day  50 Yes   12 ( 30.0%)   13 ( 35.1%)   25 ( 32.5%)   40 ( 27.0%)      Day 80 No   28 ( 70.0%)   24 ( 64.9%)   52 ( 67.5%)   108 ( 73.0%)  Total   40 (100.0%)   37 (100.0%)   77 (100.0%)   148 (100.0%)    Day 100 Yes   10 ( 26.3%)   16 ( 42.1%)   26 ( 34.2%)   44 ( 29.7%)  No   28 ( 73.7%)   22 ( 57.9%)   50 ( 65.8%)   104 ( 70.3%)  Total   38 (100.0%)   38 (100.0%)   76 (100.0%)   148 (100.0%)    Day 180 Yes   10 ( 28.6%)   11 ( 39.3%)   21 ( 33.3%)   36 ( 26.3%)  No   25 ( 71.4%)   17 ( 60.7%)   42 ( 66.7%)   101 ( 73.7%)  Total   35 (100.0%)   28 (100.0%)   63 (100.0%)   137 (100.0%)    Day 365 Yes   9 ( 32.1%)   5 ( 20.8%)   14 ( 26.9%)   28 ( 22.4%)  No   19 ( 67.9%)   19 ( 79.2%)   38 ( 73.1%)   97 ( 77.6%)  Total   28 (100.0%)   24 (100.0%)   52 (100.0%)   125 (100.0%)  -------------------------------------------------------------------------------------------------------------------  infections occurring at indicated time points in the interval to the next visit are reported. Percentages are based on total number of patients with assessment.  Suppl. Table 8: Adverse Events by Preferred Term and System Organ Class  Analysis: Safety Population  ­­­­­­­­­­­­­­­­­­­­­­­­­­­­­­­­­­­­­­­­­­­­­­­­­­­­­­­­­­­­­­­­­­­­­­­­­­­­­­­­­­­­­­­­­­­­­­­­­­­­­­­­­­­­­­­­­­  System Organ Class (SOC)/ Placebo Prednisolone Total  Preferred Term (N=45) (N=42) (N=87)  ­­­­­­­­­­­­­­­­­­­­­­­­­­­­­­­­­­­­­­­­­­­­­­­­­­­­­­­­­­­­­­­­­­­­­­­­­­­­­­­­­­­­­­­­­­­­­­­­­­­­­­­­­­­­­­­­­­  ALL SOCs  Total Pts with at Least one AE 29 ( 64.4%) 21 ( 50.0%) 50 ( 57.5%)  Total Number of AEs 60 48 108  ------------------------------------------------------------------------------------------------------------------  **GASTROINTESTINAL TRACT and LIVER AE**  Total Pts with at Least one AE 12 ( 24.4%) 8 ( 19.0%) 20 ( 21.8%)  DIARRHOEA 8 ( 17.8%) 4 ( 9.5%) 12 ( 13.8%)  NAUSEA 5 ( 11.1%) 2 ( 4.8%) 7 ( 8.0%)  VOMITING 2 ( 4.4%) 2 ( 4.8%) 4 ( 4.6%)  ABDOMINAL PAIN UPPER 1 ( 2.2%) 1 ( 2.4%) 2 ( 2.3%)  CONSTIPATION 1 ( 2.2%) 0 1 ( 1.1%)  GASTROINTESTINAL HAEMORRHAGE 1 ( 2.2%) 0 1 ( 1.1%)  OESOPHAGEAL STENOSIS 0 1 ( 2.4%) 1 ( 1.1%)  SWALLOWING DIFFICULT 1 ( 2.2%) 0 1 ( 1.1%)  VENOOCCLUSIVE DISEASE 1 ( 2.2%) 0 1 ( 1.1%)  HEPATOTOXICITY 1 ( 2.2%) 0 1 ( 1.1%)  Total Number of AEs 21 10 31  ------------------------------------------------------------------------------------------------------------------  **INFECTIONS**  Total Pts with at Least one AE 8 ( 17.8%) 7 ( 16.7%) 15 ( 17.2%)  FUNGAL INFECTIONS:  ASPERGILLOSIS 1 ( 2.2%) 1 ( 2.4%) 2 ( 2.3%)  PNEUMONIA FUNGAL 0 1 ( 2.4%) 1 ( 1.1%)  FUNGAL INFECTION 1 ( 2.2%) 0 1 ( 1.1%)  FUNGAL PHARYNGITIS 0 1 ( 2.4%) 1 ( 1.1%)  GENITAL INFECTION FUNGAL 0 1 ( 2.4%) 1 ( 1.1%)  CANDIDA INFECTION 1 ( 2.2% 3 ( 5.9%) 3 ( 2.3%)  VIRAL INFECTIONS:  ORAL HERPES 1 ( 2.2%) 1 ( 2.4%) 2 ( 2.3%))  CYTOMEGALOVIRUS INFECTION 0 1 ( 2.4%) 1 ( 1.1%)  EPSTEIN-BARR VIRAEMIA 2 ( 4.4%) 0 2 ( 2.3%)  PARAINFLUENZAE VIRUS INFECTION 1 ( 2.2%) 0 1 ( 1.1%)  GASTROENTERITIS NOROVIRUS 0 1 ( 2.4%) 1 ( 1.1%)  BACTERIALINFECTIONS:  INFECTION (not specified) 1 ( 2.2%) 0 1 ( 1.1%)  ENTEROCOCCAL INFECTION 1 ( 2.2%) 0 1 ( 1.1%)  BACTERAEMIA 1 ( 2.2%) 0 1 ( 1.1%  ESCHERICHIA BACTERAEMIA 1 ( 2.2%) 0 1 ( 1.1%)  STAPHYLOCOCCAL INFECTION 0 1 ( 2.4%) 1 ( 1.1%)  Total Number of AEs 11 10 21    **SupplTable 8 continued: Adverse Events by Preferred Term and System Organ Class**  ­­­­­­­­­­­­­­­­­­­­­­­­­­­­­­­­­­­­­­­­­­­­­­­­­­­­­­­­­­­­­­­­­­­­­­­­­­­­­­­­­­­­­­­­­­­­­­­­­­­­­­­­­­­­­­­­­­  System Organ Class (SOC)/ Placebo Prednisolone Total  Preferred Term (N=45) (N=42) (N=87)  ------------------------------------------------------------------------------------------------------------------  RENAL AND URINARY TRACT ADVERSE EVENTS  Total Pts with at Least one AE 5 ( 11.1%) 7 ( 16.7%) 12 ( 13.8%)  ACUTE KIDNEY INJURY 3 ( 6.7%) 1 ( 2.4%) 4 ( 4.6%)  HAEMATURIA: (N=3 HAEMORRHAGIC CYSTITIS) 0 4 ( 7.1%) 4 ( 3.4%)  RENAL FAILURE 1 ( 2.2%) 1 ( 2.4%) 2 ( 2.3%)  NEPHROPATHY TOXIC 1 ( 2.2%) 0 1 ( 1.1%)  NOCTURIA 0 1 ( 2.4%) 1 ( 1.1%)  Total Number of AEs 5 7 12    ------------------------------------------------------------------------------------------------------------------  GENERAL ADVERSE EVENTS AND ADMINISTRATION SITE CONDITIONS  Total Pts with at Least one AE 3 ( 6.7%) 5 ( 11.9%) 8 ( 9.2%)  MUCOSAL INFLAMMATION 0 2 ( 4.8%) 2 ( 2.3%)  OEDEMA 2 ( 4.4%) 2 ( 4.8%) 4 ( 4.6%)  OEDEMA PERIPHERAL 0 2 ( 4.8%) 2 ( 2.3%)  PYREXIA 0 1 ( 2.4%) 1 ( 1.1%)  Total Number of AEs 3 5 8    ­­­­­­­­­­­­­­­­­­­­­­­­­­­­­­­­­­­­­­­­­­­­­­­­­­­­­­­­­­­­­­­­­­­­­­­­­­­­­­­­­­­­­­­­­­­­­­­­­­­­­­­­­­­­­­­­­­  METABOLISM AND NUTRITION  Total Pts with at Least one AE 2 ( 4.4%) 3 ( 7.1%) 5 ( 5.7%)  DECREASED APPETITE 2 ( 4.4%) 0 2 ( 2.3%)  HYPERGLYCAEMIA 0 2 ( 4.8%) 2 ( 2.3%)  VITAMIN B12 DEFICIENCY 0 1 ( 2.4%) 1 ( 1.1%)  Total Number of AEs 2 3 5  ­­­­­­­­­­­­­­­­­­­­­­­­­­­­­­­­­­­­­­­­­­­­­­­­­­­­­­­­­­­­­­­­­­­­­­­­­­­­­­­­­­­­­­­­­­­­­­­­­­­­­­­­­­­­­­­­­­  RESPIRATORY, THORACIC AND MEDIASTINAL ADVERSE EVENTS  Total Pts with at Least one AE 3 ( 6.7%) 2 ( 4.8%) 5 ( 5.7%)  COUGH 1 ( 2.2%) 0 1 ( 1.1%)  DYSPNOEA 1 ( 2.2%) 0 1 ( 1.1%)  DYSPNOEA EXERTIONAL 0 1 ( 2.4%) 1 ( 1.1%)  EPISTAXIS 0 1 ( 2.4%) 1 ( 1.1%)  PULMONARY HAEMORRHAGE 1 ( 2.2%) 0 1 ( 1.1%)  CHEST PAIN 1 ( 2.2%) 0 1 ( 1.1%)  THROAT IRRITATION 0 1 ( 2.4%) 1 ( 1.1%)  Total Number of AEs 3 3 6  ------------------------------------------------------------------------------------------------------------------  CLINICAL CHEMISTRY AND VIRAL TESTING  Total Pts with at Least one AE 3 ( 6.7%) 1 ( 2.4%) 4 ( 4.6%)  C-REACTIVE PROTEIN INCREASED 2 ( 4.4%) 1 ( 2.4%) 3 ( 3.4%)  Total Number of AEs 4 1 5 -  **Suppl.Table 8 continued: Adverse Events by Preferred Term and System Organ Class**  ­­­­­­­­­­­­­­­­­­­­­­­­­­­­­­­­­­­­­­­­­­­­­­­­­­­­­­­­­­­­­­­­­­­­­­­­­­­­­­­­­­­­­­­­­­­­­­­­­­­­­­­­­­­­­­­­­­  System Organ Class (SOC)/ Placebo Prednisolone Total  Preferred Term (N=45) (N=42) (N=87)  ------------------------------------------------------------------------------------------------------------------  SKIN AND SUBCUTANEOUS TISSUE ADVERSE EVENTS  Total Pts with at Least one AE 2 ( 4.4%) 2 ( 4.8%) 4 ( 4.6%)  RASH 1 ( 2.2%) 1 ( 2.4%) 2 ( 2.3%)  ERYTHEMA 0 1 ( 2.4%) 1 ( 1.1%)  RASH PRURITIC 1 ( 2.2%) 0 1 ( 1.1%)  Total Number of AEs 2 2 4  ------------------------------------------------------------------------------------------------------------------  NERVOUS SYSTEM  Total Pts with at Least one AE 2 ( 4.4%) 1 ( 2.4%) 3 ( 3.4%)  HEADACHE 2 ( 4.4%) 0 2 ( 2.3%)  INTRACRANIAL PRESSURE INCREASED 0 1 ( 2.4%) 1 ( 1.1%)  PRESYNCOPE 0 1 ( 2.4%) 1 ( 1.1%)  Total Number of AEs 2 2 4    ------------------------------------------------------------------------------------------------------------------  PSYCHIATRIC ADVERSE EVENTS  Total Pts with at Least one AE 1 ( 2.2%) 1 ( 2.4%) 2 ( 2.3%)  CONFUSION 0 1 ( 2.4%) 1 ( 1.1%)  HALLUCINATION 0 1 ( 2.4%) 1 ( 1.1%)  INSOMNIA 1 ( 2.2%) 0 1 ( 1.1%)  Total Number of AEs 1 2 3  ------------------------------------------------------------------------------------------------------------------  PROLONGED APLASIA  Total Pts with at Least one AE 2 ( 4.4%) 0 2 ( 2.3%)  APLASIA 2 ( 4.4%) 0 2 ( 2.3%)  Total Number of AEs 2 0 2  ------------------------------------------------------------------------------------------------------------------  VASCULAR DISORDERS  Total Pts with at Least one AE 1 ( 2.2%) 1 ( 2.4%) 2 ( 2.3%)  INTERMITTENT CLAUDICATION 0 1 ( 2.4%) 1 ( 1.1%)  Total Number of AEs 1 1 2  ------------------------------------------------------------------------------------------------------------------  BLOOD AND LYMPHATIC SYSTEM DISORDERS  Total Pts with at Least one AE 1 ( 2.2%) 0 1 ( 1.1%)  PANCYTOPENIA 1 ( 2.2%) 0 1 ( 1.1%)  Total Number of AEs 1 0 1  ------------------------------------------------------------------------------------------------------------------  EYE DISORDERS  Total Pts with at Least one AE 0 1 ( 2.4%) 1 ( 1.1%)  CATARACT 0 1 ( 2.4%) 1 ( 1.1%)  Total Number of AEs 0 1 1    Table 9 TSAE_S: Serious Adverse Events by Preferred Term and System Organ Class  Protocol: PRE-GvHD (EudraCT: 2008-005862-30)  Analysis: Safety Population  ­­­­­­­­­­­­­­­­­­­­­­­­­­­­­­­­­­­­­­­­­­­­­­­­­­­­­­­­­­­­­­­­­­­­­­­­­­­­­­­­­­­­­­­­­­­­­­­­­­­­­­­­­­­­­­­­­­  System Organ Class (SOC)/ Placebo Prednisolone Total  Preferred Term (N=45) (N=42) (N=87)  ­­­­­­­­­­­­­­­­­­­­­­­­­­­­­­­­­­­­­­­­­­­­­­­­­­­­­­­­­­­­­­­­­­­­­­­­­­­­­­­­­­­­­­­­­­­­­­­­­­­­­­­­­­­­­­­­­­    ALL SOCs  Total Pts with at Least one AE 6 ( 13.3%) 3 ( 7.1%) 9 ( 10.3%)  Total Number of AEs 8 4 12    GASTROINTESTINAL TRACT  Total Pts with at Least one AE 3 ( 6.7%) 1 ( 2.4%) 4 ( 4.6%)  DIARRHOEA 1 ( 2.2%) 1 ( 2.4%) 2 ( 2.3%)  GASTROINTESTINAL HAEMORRHAGE 1 ( 2.2%) 0 1 ( 1.1%)  NAUSEA 1 ( 2.2%) 0 1 ( 1.1%)  SWALLOWING DIFFICULT 1 ( 2.2%) 0 1 ( 1.1%)  VOMITING 1 ( 2.2%) 0 1 ( 1.1%)  Total Number of AEs 5 1 6    BLOOD AND LYMPHATIC SYSTEM  Total Pts with at Least one AE 1 ( 2.2%) 0 1 ( 1.1%)  PANCYTOPENIA 1 ( 2.2%) 0 1 ( 1.1%)  Total Number of AEs 1 0 1        INFECTIONS AND INFESTATIONS  Total Pts with at Least one AE 0 1 ( 2.4%) 1 ( 1.1%)  ORAL HERPES 0 1 ( 2.4%) 1 ( 1.1%)  PNEUMONIA FUNGAL 0 1 ( 2.4%) 1 ( 1.1%)  Total Number of AEs 0 2 2    ------------------------------------------------------------------------------------------------------------------          Investigator text for Adverse Events encoded using MedDRA version 19.1.  Percentages are based on N.  Multiple occurrences of the same adverse event in one individual counted only once.  Unrelated adverse events with onset before start of pre-emptive therapy are excluded. |
| --- |

**Supplemental Table 9 Pilot study**

| ^†^Mann-Whitney-Test |  |
| --- | --- |
| * 2 pts no status, non-malignant |  |
| **TCD: T-cell depletion; no immunosuppression | |

**Suppl. Table 9 Pilot study** summarizes the pilot patients from our history study. Here matched pairs of patients receiving prednisolone upon the positivity of the first sample for the aGvHD_MS17 test. The control group was matched as closely as possible for age, for primary disease, donor, HLA-match and conditioning regimen. The outcome was significantly more patients without aGvHD in the pre-emptive prednisolone group compared to the control group. **Abbreviations Suppl. Table 10**: acute: AML: acute myeloid leukemia; ALL: acute lymphatic leukemia; sAML: secondary AML; chronic: MDS/MPS: myelodysplastic/proliferative syndrome; CML: chronic myeloid leukemia; non-malignant: SAA: severe or very severe aplastic anemia; CR/CP complete remission/ chronic phase; no CR: untreated, relapse, refractory; MAC myeloablative conditioning RIC: reduced intensity conditioning; PBSC: peripheral blood stem cells; BM: bone marrow; CB: cord blood; ATG: anti-thymocyte globulin: CSA: cyclosporine A; MTX: methotrexate; MMF: mycophenolate motefil; other: MMF, tacrolimus (FK506), or different combinations of immunosuppressants.

**Suppl Table 10: Comparison of conditioning regimen of previously published cohort ^5^ and the Pre-GvHD patient cohort:**

**Suppl Table 10** shows the comparison of conditioning protocols and immunosuppressive antibodies for the “Pre-GvHD”- and a previously published patient cohort (“Leukemia”) ^5^. In our first large patient cohort the majority of the FLAMSA protocol was the major RIC. FLAMSA was designed to allow for GvHD development in order to treat patients with refractory disease and patients in relapse with or without follow-up donor leukocyte transfusion (DLI). Abbreviations: Fludarabin (Flu), Amsacrine, AraC and TBI or Busilvex (FLAMSA ^6^); FBM: Flu-Busulfane, Melphalane; BNCU-Flu-Melphalane (BFM); Flu-Treosulfane (Flu-Treo); total body irradiation (TBI)-Flu and other.

1. Weissinger EM, Nguyen-Khoa T, Fumeron C, Saltiel C, Walden M, Kaiser T*, et al.* Effects of oral vitamin C supplementation in hemodialysis patients: A proteomic assessment. *Proteomics* 2006 Feb; **6**(3)**:** 993-1000.

2. Weissinger EM, Wittke S, Kaiser T, Haller H, Bartel S, Krebs R*, et al.* Proteomic patterns established with capillary electrophoresis and mass spectrometry for diagnostic purposes. *Kidney International* 2004 Jun; **65**(6)**:** 2426-2434.

3. Weissinger EM, Mischak H, Kontsendorn J, Hahn A, Hahn N, Morgan M*, et al.* Proteome analysis in hematology using capillary electrophoresis coupled on-line to mass spectrometry. *Mini reviews in medicinal chemistry* 2009 May; **9**(5)**:** 627-623.

4. Mischak H, Coon JJ, Novak J, Weissinger EM, Schanstra JP, Dominiczak AF. Capillary electrophoresis-mass spectrometry as a powerful tool in biomarker discovery and clinical diagnosis: an update of recent developments. *Mass spectrometry reviews* 2009 Sep-Oct; **28**(5)**:** 703-724.

5. Weissinger EM, Metzger J, Dobbelstein C, Wolff D, Schleuning M, Kuzmina Z*, et al.* Proteomic peptide profiling for preemptive diagnosis of acute graft-versus-host disease after allogeneic stem cell transplantation. *Leukemia* 2014 Apr; **28**(4)**:** 842-852.

6. Schmid C, Schleuning M, Schwerdtfeger R, Hertenstein B, Mischak-Weissinger E, Bunjes D*, et al.* Long-term survival in refractory acute myeloid leukemia after sequential treatment with chemotherapy and reduced-intensity conditioning for allogeneic stem cell transplantation. *Blood* 2006 Aug 1; **108**(3)**:** 1092-1099.
